# Developmental staging models in bipolar disorder

**DOI:** 10.1186/s40345-015-0033-1

**Published:** 2015-07-31

**Authors:** Ives C Passos, Karen Jansen, Flavio Kapczinski

**Affiliations:** UT Center of Excellence on Mood Disorder, Department of Psychiatry and Behavioral Sciences, The University of Texas Science Center at Houston, 1941 East Road, Houston, TX 77054 USA; Bipolar Disorder Program and Laboratory of Molecular Psychiatry, Federal University of Rio Grande do Sul, Porto Alegre, RS Brazil; Health and Behavior Program, Catholic University of Pelotas, Pelotas, RS Brazil

**Keywords:** Bipolar disorder, Staging models, Prodromal symptoms

## Abstract

The previous contribution of Duffy and colleagues suggests that a chain of behavioral events starting during childhood precedes the development of full-blown bipolar disorder. In this vein, the recent contribution of Keown-Stoneman and colleagues brings a new perspective to the study of prodromal symptoms of bipolar disorder.

## Findings

**Fig. 1:**
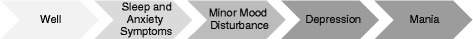
Course of prodromal symptoms in bipolar disorder

In 2010, Duffy et al. showed that subjects diagnosed with bipolar disorder (BD) followed a predictable sequence of prodromal symptoms (Duffy et al. 2010). Specifically, the majority of premorbid children developed prodromal symptoms including sleep and anxiety disturbances which evolved to minor depressive symptoms and sensitivity to stress during puberty (Duffy et al. 2010). Subsequently, in mid-adolescence, recurrent major depressive episodes occurred. Around 3 years later, conversion to BD took place with the emergence of hypomanic/manic episodes (Duffy et al. 2010) (Fig. 1). Duffy et al. also showed that combining familial risk with the presence of an anxiety disorder elevated the age-adjusted risk for a major mood disorder to over 80 % (Duffy et al. 2010). This is an interesting finding, since whereas a positive family history of BD is the strongest risk factor for bipolar disorder (Lapalme et al. 1997), the majority of first-degree relatives of bipolar probands do not develop bipolar disorder (Rice et al. 1987). Of note, it also recognized that not all offspring go through all stages, rather once an individual joins the staging model, they progress from that point forward (i.e., may skip stage 1 and enter at stage 2 jumping from well to minor mood/adjustment disorders). Within this current issue, Keown-Stoneman and colleagues moved the field forward showing the estimated probability of patients being in each stage of the model previously described (Duffy et al. 2010).

Generally, staging models imply that natural history of the disorder moves through a predictable temporal progression, and provision of stage-appropriate treatment can modify such course. Some medical specialties such as cardiology and oncology have advanced in this field, developing strategies to prevent both the onset and the development of established disease. In the same way, bipolar disorder identified in the early stages may be less treatment refractory with a greater probability of response to monotherapy (Scott et al. 2006; Swann et al. 1999). Therefore, the contribution of developmental staging is really important since it provides the opportunity to intervene therapeutically in premorbid patients with bipolar disorder thus preventing a more pernicious course of illness.

In 2007, Berk et al. presented a staging model of the longitudinal course of bipolar disorder and the temporal impact of interventions (Berk et al. 2007). Subjects at stage 0 are those at increased risk of severe mood disorder (e.g., family history). Subjects with prodromal features of bipolar disorder were classified at stage 1, whereas those who had the first-episode threshold mood disorder were classified in stage 2. Subjects at stage 3 were those with recurrent mood episodes, and subjects with persistent unremitting illness were classified at stage 4. The model emphasizes early detection and algorithm appropriate intervention where possible. The rationale for this model is that early intervention is likely to be associated with a better response to treatment.

In the same vein, Kapczinski et al. presented a staging model that emphasizes the assessment of patients in the inter-episodic period and includes the following: latent phase—individuals who present mood and anxiety symptoms and increased risk for developing threshold bipolar disorder; stage I—patients with bipolar disorder who present well-established periods of euthymia and absence of overt psychiatric morbidity between episodes; stage II—patients who present rapid cycling or current axis I or II comorbidities; stage III—patients who present a clinically relevant pattern of cognitive and functioning deterioration, as well as altered biomarkers; and stage IV—patients who are unable to live autonomously and present altered brain scans and biomarkers (Kapczinski et al. 2009). The progression across these presentations may be engendered by the cumulative exposure to acute episodes, drug abuse, life stress, and inherited vulnerability (Kapczinski et al. 2009). As a corollary of these early descriptions, the notion of functional staging emerged in the field of bipolar disorder. Functional staging may provide a practical model to guide therapy to improve quality of life. In this regard, a strong linear association was reported between The Functioning Assessment Short Test (FAST) scores and the clinical stages described by Kapczinski (Kapczinski et al. 2009), suggesting a progressive functional decline from stage I through stage IV of bipolar disorder (Rosa et al. 2014). Similarly, by applying latent class analysis in a sample of 106 remitted adults with bipolar disorder, one study identified two subtypes of patients presenting “good” and “poor” functional outcomes (Reinares et al. 2013). Estimated verbal intelligence, inhibitory control, episode density, and level of residual depressive symptoms emerged as the most significant predictors of each subtype (Reinares et al. 2013). Of note, functional outcome was not predicted by illness duration, since the two groups were comparable in age, age of onset, and illness duration (Reinares et al. 2013).

Moreover, a recent task force of the International Society for Bipolar Disorders has performed a systematic review on staging models for bipolar disorders (Kapczinski et al. 2014). It showed that available data converge to suggest that broadly defining bipolar disorder as early and late stage is heuristically useful (Kapczinski et al. 2014). Also, the review has shown a proposal for functional neuroimaging findings according staging in 2014 (Frank et al. 2014). The structural changes were categorized in two themes: emotion processing and reward processing (Frank et al. 2014). Resilience and risk markers for individuals at risk and markers of disease progression for patients with bipolar disorder were also stated in the study (Table 1).

**Table 1:** Neuroimaging findings and staging models of bipolar disorder

Clinical staging	Clinical presentation	Neuroimaging findings
0	Increased risk of bipolar disorder; no symptoms currently	*Resilience markers:* abnormal prefrontal cortical activity increases during cognitive control of emotion and cognitive control tasks; abnormal volumetric increases in right-sided vlPFC and left-sided subcortical regions
		*Risk markers:* abnormally increased amygdala activity; abnormal prefrontal WM
1	a) Mild or nonspecific symptoms	*Resilience markers:* Abnormally increased prefrontal cortical activity during cognitive control of emotion and cognitive control tasks; abnormally increased prefrontal cortical volume
	b) Ultra-high-risk: moderate but subthreshold symptoms, with neurocognitive changes and functional decline to caseness	*Risk markers:* abnormally decreased prefrontal cortical volumes; left-sided subcortical volume increases; abnormally decreased WM volume
2	First episode of bipolar disorder; full threshold disorder with moderate to severe symptoms, neurocognitive deficits, and functional decline	*Emotion processing:* abnormally decreased prefrontal cortical activity (especially right-sided vlPFC activity) during cognitive control of emotion and cognitive control tasks; abnormally increased amygdala activity during these tasks; abnormally decreased prefrontal cortical volumes and decreased prefrontal WM; altered subcortical volumes
		*Reward processing:* abnormally increased left-sided striatal and prefrontal cortical activity during reward processing
3	a) Incomplete remission from first episode (could be linked or fast-tracked to stage 4)	*Markers of disease progression:* a negative association between prefrontal cortical volumes (especially right vlPFC gray matter volume) and illness duration; reductions in amygdala, striatal, and hippocampal volumes with illness progression
	b) Recurrence or relapse of psychotic or mood disorder which stabilizes with treatment, residual symptoms, or neurocognition below the best level achieved following remission from first episode	
4	Severe, persistent illness as judged on symptoms, neurocognition, and disability criteria	

Adapted from Frank E et al. (Frank et al. 2014)

*vlPFC* ventrolateral prefrontal cortex, *WM* white matter

In conclusion, the current paper (Keown-Stoneman et al. 2015) and the previous studies of Duffy and colleagues have created a heuristic model for staging prodromal phases of bipolar disorder. This added to the notion that bipolar disorder may be conceived in terms of differential stages (Duffy et al. 2010). Of note, we need to use the specific evidence pertaining to BD from longitudinal studies of high-risk individuals and patients to develop a comprehensive specific staging model for BD. The opportunity to characterize transitions to full-blown illness and developmental staging models is the first step to paradigm shifts in preventative strategies. Recent progress in molecular psychiatry and the use of multiscale datasets coupled with machine learning techniques may help to refine predictive models of conversion to bipolar disorder in clinical and population samples (Kapczinski and Passos 2015).
